# GABAergic Network Dysregulation in Alcohol Use Disorder: Receptor–Transporter Interactions Driving the Transition From Goal‐Directed to Compulsive Behaviour

**DOI:** 10.1111/adb.70195

**Published:** 2026-10-29

**Authors:** Hannah Aranda, Nathan Jones, Humza Haroon, Doo‐Sup Choi

**Affiliations:** ^1^ Department of Molecular Pharmacology and Experimental Therapeutics Mayo Clinic College of Medicine and Science Rochester Minnesota USA; ^2^ Department of Psychiatry and Psychology Mayo Clinic College of Medicine and Science Rochester Minnesota USA

## Abstract

Alcohol use disorder (AUD) is characterized by a shift from controlled, goal‐directed drinking to compulsive, habitual alcohol (a.k.a. ethanol) seeking, even in the face of negative outcomes. This transition is closely associated with alterations in cortico‐striatal circuitry and dysregulated GABAergic signalling, both of which drive the progression from flexible control to compulsive behaviour. Acute alcohol exposure enhances GABAergic inhibition, primarily by increasing presynaptic GABA release. Chronic exposure leads to compensatory neuroadaptations, such as subunit reorganization, receptor internalization and diminished GABA receptor sensitivity. While these neuroadaptations alter receptor‐mediated GABAergic signalling during dependence and withdrawal, they also alter dopaminergic regulation and further reinforce alcohol‐seeking behaviour. Beyond receptor‐level adaptations, impaired GABA clearance, especially through the astrocytic transporter GAT3, plays a crucial role in regulating extracellular GABA concentrations and tonic inhibition. This effect is particularly evident within the dorsal striatum, where elevated tonic GABA shifts control from flexible, goal‐directed action towards rigid, habitual responding by altering the balance between the dorsomedial and dorsolateral subregions. Consistent with these mechanistic insights, animal and human studies demonstrate that reduced GAT3 expression is associated with compulsive alcohol use and impaired reward‐related behaviour, reinforcing impaired GABA clearance as a central mechanism in this behavioural shift. Taken together, these lines of evidence validate a central therapeutic model, namely, that targeting altered tonic inhibition and impaired GABA clearance in AUD could address the inhibitory dysfunction that sustains the disorder. Overall, this work supports a unifying model in which receptor adaptations and disrupted GABA removal maintain altered inhibitory tone, driving maladaptive circuit function and contributing to the persistence of AUD.

## Graphical abstract

Astrocytic GAT3 regulates ambient GABA levels in the globus pallidus externus (GPe). Reduced GAT3 function enhances extrasynaptic GABAergic signalling and disrupts subthalamic nucleus (STN) regulation and cortical signal fidelity.

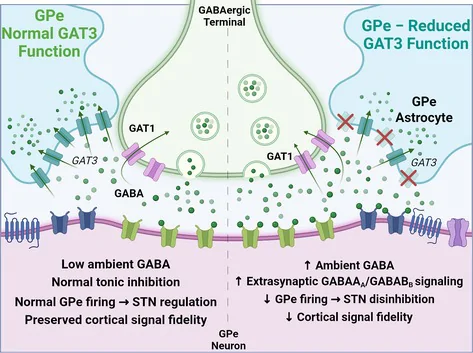

## 1 Introduction

Substance use disorders (SUDs), including alcohol use disorder (AUD), demonstrate how neurobiological changes can undermine behavioural control by disrupting action‐outcome contingency. This process allows behaviour to be flexibly updated when an action no longer produces its expected outcome [1]. This disruption extends beyond habitual seeking and may help explain persistent substance use despite adverse consequences, a defining feature of addiction [2, 3]. In the United States, the National Survey on Drug Use and Health (NSDUH) reported that in 2024, 46.6% of people aged 12 or older used alcohol in the past month, and 43.1% of them met criteria for binge drinking. Moreover, 10.8% met criteria for heavy alcohol use [4]. However, heavy alcohol use, such as binge drinking, is not sufficient by itself to diagnose AUD.

Alcohol use disorder (AUD), as defined in the DSM‐5, is characterized by impaired control over alcohol use, increased priority given to drinking, risky use and physiological adaptation, including tolerance or withdrawal. Clinicians diagnose AUD when an individual meets at least two of 11 criteria within 12 months; severity is classified as mild (two to three symptoms), moderate [4, 5] or severe (six or more) [5]. According to the 2024 NSDUH, 27.9 million people aged 12 or older in the US met criteria for AUD [4]. More broadly, AUD reflects a fundamental disruption in behavioural control, marked by a shift from goal‐directed to habitual alcohol seeking. This impaired control defines the feature of AUD.

Goal‐directed behaviour relies on action‐outcome contingency, whereby actions are selected and flexibly modified based on their expected outcomes. In contrast, habitual behaviours persist even as outcome value changes, with actions becoming increasingly dissociated from their consequences and behavioural control shifting towards stimulus–response associations [6, 7]. This review focuses on how disrupted GABAergic signalling may shift alcohol seeking from flexible, goal‐directed control to compulsive and inflexible patterns. While alcohol use may be initially goal‐directed, over time it can become more habitual, exhibiting outcome‐insensitive responding and ultimately progressing to compulsive use despite negative health and social consequences [8, 9]. Value‐devaluation paradigms help differentiate these seeking behaviours [10]. However, in human studies, alcohol dependence results from a complex interplay of factors beyond simple action‐outcome or stimulus–response associations, and it would be an oversimplification to characterize AUD solely as goal‐directed, habitual or compulsive behaviour [11]. Instead, AUD may arise from the convergence of these processes.

Recent evidence indicates that impaired clearance of extracellular gamma‐aminobutyric acid (GABA) can disrupt inhibitory signalling and shift the balance between flexible and habitual responding. Reduced GABA uptake, particularly via transporters such as gamma‐aminobutyric acid transporter 3 (GAT3), elevates extracellular GABA and may disturb excitatory and inhibitory signalling in brain regions implicated in reward processing, habit formation and stress responsiveness (see Table 1 for nomenclature of GABA transporters). In this way, altered GABA homeostasis may contribute to compulsive alcohol seeking and impaired behavioural flexibility [12, 13]. Alcohol‐induced changes in receptor subunit composition, localization and function can further reshape neural circuits, linking these molecular adaptations to compulsive alcohol seeking and impaired flexibility [14, 15, 16]. While this review focuses on GABAergic signalling, glutamatergic circuits also play a crucial role in the shift from goal‐directed to habitual control. Excitatory input to the striatum, particularly via cortico‐striatal and amygdalo‐striatal projections, helps determine which behavioural strategy dominates. These glutamatergic pathways provide a major mechanism by which alcohol biases control toward habitual responding. For example, orbitofrontal projections to the dorsal striatum are necessary for goal‐directed action control, and endocannabinoid‐dependent attenuation of this pathway enables the shift towards habitual behaviour [17]. Chronic intermittent alcohol exposure reduces the excitability of orbitofrontal circuits that support goal‐directed control, and selective weakening of orbitofrontal output to the dorsomedial striatum's direct pathway promotes habitual control. Enhancing activity in these cortical circuits can restore goal‐directed behaviour [18]. In parallel, the basolateral amygdala's glutamatergic activity tracks and mediates the incentive value required for goal‐directed actions, with support from dissociable orbitofrontal inputs [19, 20]. Excessive alcohol consumption potentiates glutamatergic transmission at both cortico‐striatal and amygdalo‐striatal inputs to the dorsomedial striatum, through postsynaptic and presynaptic mechanisms, respectively [21]. Collectively, these findings show that alcohol remodels excitatory drive within behavioural circuits, promoting the shift from goal‐directed to habitual responding.

**TABLE 1:** Nomenclature of GABA transporters and related terms used.

Term used in review	Gene name (human)	Alternative name(s)	Primary cellular localization
GAT1	*SLC6A1*	GABA transporter 1	Predominantly neurons (presynaptic terminals)
GAT2	*SLC6A13*	GABA transporter 2	Liver, kidney, meninges, limited CNS expression
GAT3	*SLC6A11*	GABA transporter 3	Astrocytes, especially distal astrocytic processes
BGT1	*SLC6A12*	Betaine/GABA transporter 1	Low CNS expression; peripheral tissues
vGAT	*SLC32A1*	Vesicular GABA transporter	Presynaptic vesicles
GAD65	*GAD2*	Glutamate decarboxylase 65	GABAergic neurons
GAD67	*GAD1*	Glutamate decarboxylase 67	GABAergic neurons
Phasic inhibition	—	Synaptic inhibition	Synaptic GABA_A_ receptors
Tonic inhibition	—	Extrasynaptic inhibition	Extrasynaptic GABA_A_ receptors
GABA_A_ receptors	Multiple genes	Ionotropic GABA receptors	Synaptic and extrasynaptic membranes
GABA_A_ receptors	*GABBR1, GABBR2*	Metabotropic GABA receptors	Pre‐ and postsynaptic compartments

*Note:* This table lists abbreviations, gene/protein names and key terms used in the text for clarity and consistency.

Building on this, the review examines receptor‐specific effects, transporter dynamics and circuit‐level consequences of alcohol exposure within GABAergic systems to support a central model: Disrupted inhibitory signalling drives compulsive alcohol seeking and impaired behavioural flexibility in AUD. Our central model is that altered tonic inhibition and impaired GABA clearance sustain this inhibitory dysfunction, and we propose that targeting these changes could address the mechanisms maintaining the disorder. Overall, this work supports a unifying model in which receptor adaptations and disrupted GABA removal maintain altered inhibitory tone and the persistence of AUD.

## 2 GABAergic Signalling in Goal‐Directed and Habitual Alcohol‐Seeking Behaviours

### 2.1 Alcohol Exposure and Cellular Sources of GABAergic Signalling

Understanding this relationship requires first considering GABA biology, as GABAergic signalling is the central mechanism linking alcohol exposure to subsequent changes in inhibitory control. GABA is the primary inhibitory neurotransmitter in the central nervous system (CNS) [22]. Its synthesis occurs through interconnected neuronal and astrocytic pathways, reflecting the complementary roles of these cell types in maintaining inhibitory tone within the CNS [23, 24, 25]. Because GABA mediates inhibition at an estimated 30%–40% of all synapses, precise regulation of its synthesis is essential for proper inhibitory control and the behavioural changes that follow alcohol exposure. The highest concentrations of GABA are found in the substantia nigra (SNc), followed by the globus pallidus (GP) and nucleus accumbens (NAc) [26]. In neurons, GABA is primarily produced from glutamate via glutamic acid decarboxylase (GAD), which exists in two main isoforms: GAD65, which supports activity‐dependent synthesis, and GAD67, which contributes to basal production [27, 28]. Both enzymes are sensitive to alcohol exposure (see Table 2), linking alcohol‐induced changes in GABA synthesis to alterations in inhibitory control and the behavioural responses that follow. As we illustrated in Figure 1, once synthesized, neuronal GABA is packaged into synaptic vesicles by the vesicular GABA transporter (vGAT) and released into the synaptic cleft during inhibitory neurotransmission, shaping downstream inhibitory signalling and influencing behavioural output [27, 28]. While neuronal GABA synthesis primarily supports vesicular release, astrocytes also play a crucial role in GABA homeostasis through alternative metabolic pathways that regulate neurotransmitter recycling and provide metabolic support [36, 37]. Astrocytes can convert glutamate to GABA via GAD, though this pathway operates at lower levels under physiological conditions. They also control extracellular GABA availability through transporter‐mediated uptake, primarily via GAT3 [38, 39, 40, 41, 42]. Additionally, astrocytes generate GABA through polyamine degradation, most notably by metabolizing putrescine via monoamine oxidase B (MAO‐B)‐dependent pathways. Unlike canonical neuronal release, astrocytes typically release GABA not through classical vesicular mechanisms; instead, they can release it tonically via channels or transporters, contributing to ambient extracellular GABA levels and tonic inhibition. By shaping tonic inhibition, astrocytes influence the inhibitory environment and the behavioural responses it can bias, including alcohol‐related behaviours [43].

**TABLE 2:** GABAergic enzymes and their association with AUD.

Enzyme	Gene	Association with AUD	References
Glutamic acid decarboxylase 1 (GAD67)	*GAD1*	Altered GAD1 expression reported in post‐mortem brain tissue of individuals with AUD, indicating disrupted GABA synthesis.	[29, 30, 31]
Glutamic acid decarboxylase 2 (GAD65)	*GAD2*	Polymorphisms in GAD2 associated with alcohol dependence and behavioural traits related to addiction (e.g., impulsivity, reward sensitivity).	[29, 30, 32, 33]
Monoamine oxidase B (MAO‐B)	*MAOB*	Reduced MAO‐B activity consistently observed in individuals with alcohol dependence; PET imaging and biochemical studies show decreased enzyme levels in brain and platelets. No observed genetic association, primarily biochemical/enzymatic activity evidence	[29]
Aldehyde dehydrogenase 1A1 (ALDH1A1)	*ALDH1A1*	Variants in ALDH family genes, including ALDH1A1, are associated with alcohol metabolism and risk for AUD; indirect but human genetic association present	[29, 34, 35]

**FIGURE 1:**
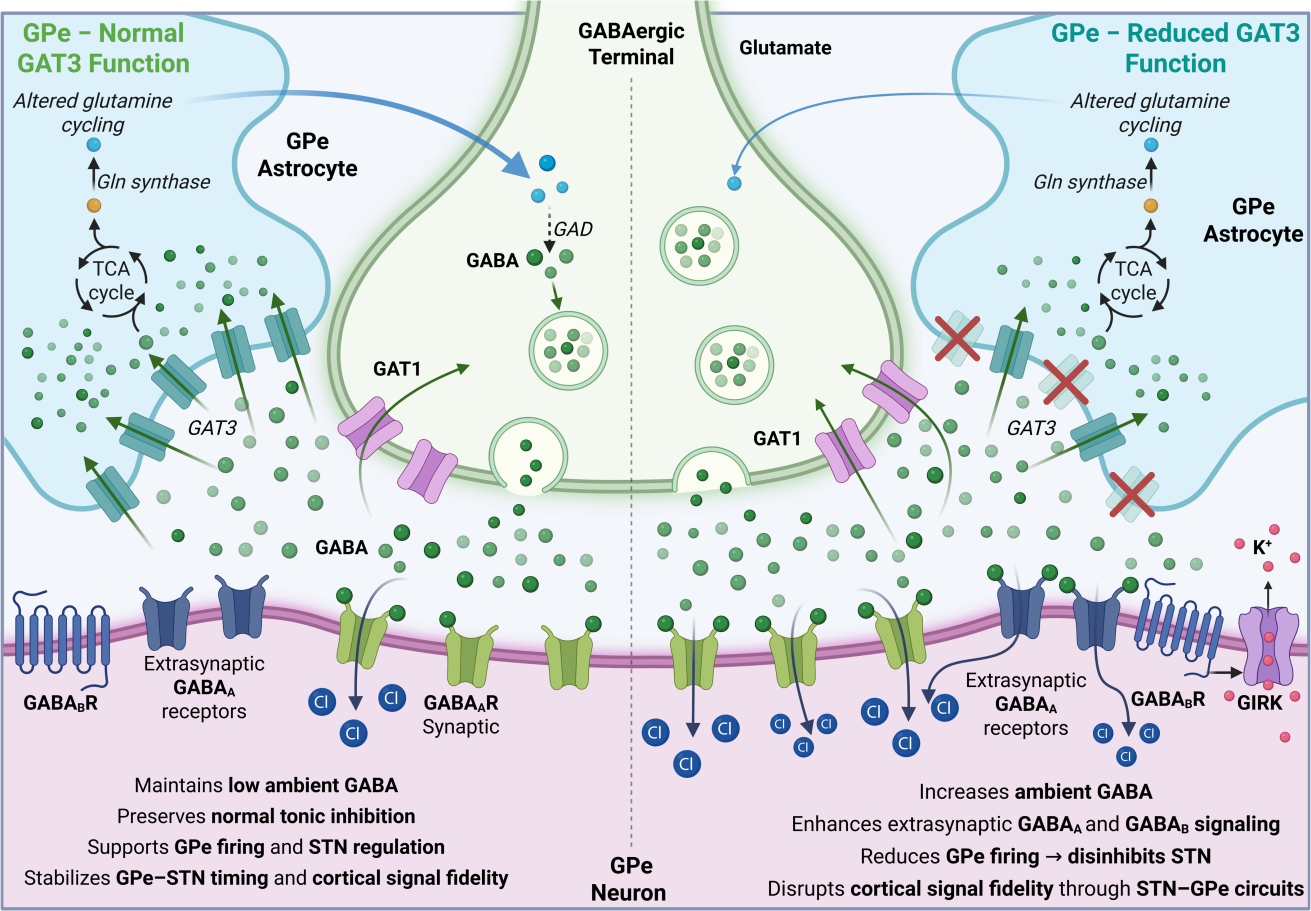
Dysregulation of astrocytic GAT3 function impairs GABA clearance and GABA receptor‐mediated signalling.

### 2.2 Phasic and Tonic Inhibition in Striatal Control of Goal‐Directed and Habitual Behaviour

Together, neuronal and astrocytic GABA sources form a tightly regulated system that coordinates phasic and tonic inhibition. Here, we focus on how this system contributes to goal‐directed and habitual alcohol‐seeking behaviours [44, 45], an area that requires further elucidation. Phasic inhibition refers to rapid, transient synaptic signalling, mediated by vesicular GABA release from presynaptic neurons and subsequent activation of GABA_A_ receptors [46]. Operating on a millisecond timescale, phasic inhibition provides precise temporal control over neuronal firing and is thought to support action selection and action‐outcome learning [45, 46]. In contrast, tonic inhibition arises predominantly from ambient extracellular GABA, originating from synaptic spillover and astrocytic release, and primarily activates extrasynaptic GABA_A_ receptors [48, 49]. By shifting the balance between these forms of inhibition, GABA signalling can alter the circuitry that supports goal‐directed and habitual alcohol‐seeking and the behaviour that follows.

A key question is how altered GABAergic tone in striatal circuits contributes to compulsive alcohol seeking by shifting control from goal‐directed to habitual responding. The striatum is especially relevant, as altered inhibitory tone here may drive the transition from flexible, outcome‐sensitive actions to rigid, outcome‐insensitive responding in AUD [6, 50, 51, 52, 53]. As the basal ganglia's main input structure, the striatum serves as a critical hub where changes in inhibitory signalling can reshape behavioural strategy and influence action selection. This transition is tightly linked to cortico‐striatal circuitry, where cortical inputs integrate within basal ganglia loops to guide action selection. While other brain regions, such as the prefrontal cortex and amygdala, contribute to decision‐making and emotion, the striatum uniquely integrates action‐outcome (A‐O) and stimulus–response (S‐R) contingencies that define goal‐directed and habitual control [54, 55, 56, 57]. The dorsal striatum comprises functionally distinct subregions: the dorsomedial striatum (DMS), dorsolateral striatum (DLS) and tail of the striatum (TS). Through distinct cortical input patterns to direct and indirect basal ganglia pathways, these subregions differentially contribute to flexible versus rigid behavioural control [40, 58, 59, 60]. Thus, focusing on striatal circuits provides a precise mechanistic link between altered GABAergic signalling, particularly changes in tonic inhibition, and the behavioural shifts underlying compulsive alcohol seeking.

### 2.3 Tonic GABA Signalling and Striatal Dysregulation of Behavioural Flexibility

Because over 90% of striatal neurons are GABAergic, their activation releases GABA onto downstream targets [61, 62, 63], rendering the dorsal striatum highly sensitive to extracellular GABA levels and inhibitory tone. This makes it an ideal region for examining how disrupted GABA regulation shifts behavioural control from goal‐directed to habitual responding. Within the dorsal striatum, a key distinction exists between the dorsomedial striatum (DMS) and dorsolateral striatum (DLS), which preferentially support goal‐directed and habitual behaviours, respectively [51, 64, 65]. These region‐specific effects are further shaped by striatal output through direct (D1‐receptor‐expressing) and indirect (D2‐receptor‐expressing) pathways: The direct pathway promotes action by disinhibiting downstream targets, while the indirect pathway suppresses action via complementary inhibitory circuits [66, 67]. By influencing the baseline excitability of these pathways, tonic GABA can alter the balance of direct and indirect output, thereby modulating how DMS and DLS circuits regulate action selection and behavioural flexibility [68, 69].

In the DMS, moderate tonic inhibition supports balanced integration of these pathways, enabling flexible, goal‐directed behaviour by allowing circuits to respond dynamically to changing action‐outcome contingencies [70, 71, 72, 73, 74, 75]. Excessive alcohol consumption can disrupt this balance by inducing long‐term potentiation of glutamatergic transmission at corticostriatal synapses onto D2‐ and D1‐receptor‐expressing medium spiny neurons [76]. This enhanced excitation may shift corticostriatal circuits towards maladaptive alcohol seeking, promoting persistent behaviour despite negative outcomes and linking the synaptic mechanism more directly to AUD [77].

In the DLS, elevated tonic inhibition may destabilize habit‐promoting circuits by dampening sensitivity to outcome feedback and reinforcing repeated, automatic actions [47, 75, 78]. More broadly, tonic GABA constrains the dopamine signal these circuits receive, directly linking this mechanism to habitual control by reducing the dopaminergic input that supports habit‐related behaviour. In the mouse dorsal striatum, ambient GABA acting at GABA_A_ and GABA_B_ receptors tonically inhibits dopamine release [79], reducing dopaminergic input and influencing habit formation. Blockade of GAT1 or GAT3 reduces evoked dopamine in the DLS but not in the NAc core, without changing striatal dopamine content, indicating a release‐specific effect. Metabolic inactivation of astrocytes abolishes this effect, implicating astrocytic GABA uptake in dopamine regulation. Together, these findings suggest that astrocytic GABA uptake in the DLS is a key constraint on habitual control, as it regulates both output pathway excitability and dopaminergic input, shapes habitual behaviour and directly links GAT3 to the regulation of habitual actions.

Alcohol also remodels phasic inhibition within the DLS, specifically at parvalbumin‐expressing (PV+) fast‐spiking interneurons (FSIs), distinct from the projection neurons discussed above. Chronic intermittent alcohol exposure reduces GABAergic, but not glutamatergic, transmission onto FSIs. This reduction reflects a loss of GABAergic synapses on FSI somata and proximal dendrites, rather than postsynaptic silencing, since responses to photo‐uncaged GABA are preserved [80]. This synaptic loss affects inhibitory inputs from both the globus pallidus and the reticular thalamic nucleus (Figure 2). It is accompanied by degradation of the perineuronal nets that surround FSIs, evidenced by fewer perineuronal net‐positive FSIs and reduced striatal aggrecan and HAPLN1 protein. Enzymatic digestion of perineuronal nets mimics the reduction in GABAergic transmission onto FSIs, and silencing pallidal and thalamic inhibitory input to the DLS increases voluntary alcohol consumption without affecting sucrose intake. Together, these findings show that alcohol weakens GABAergic inputs and degrades perineuronal nets surrounding FSIs, reshaping inhibitory signalling in the DLS and increasing voluntary alcohol consumption.

**FIGURE 2:**
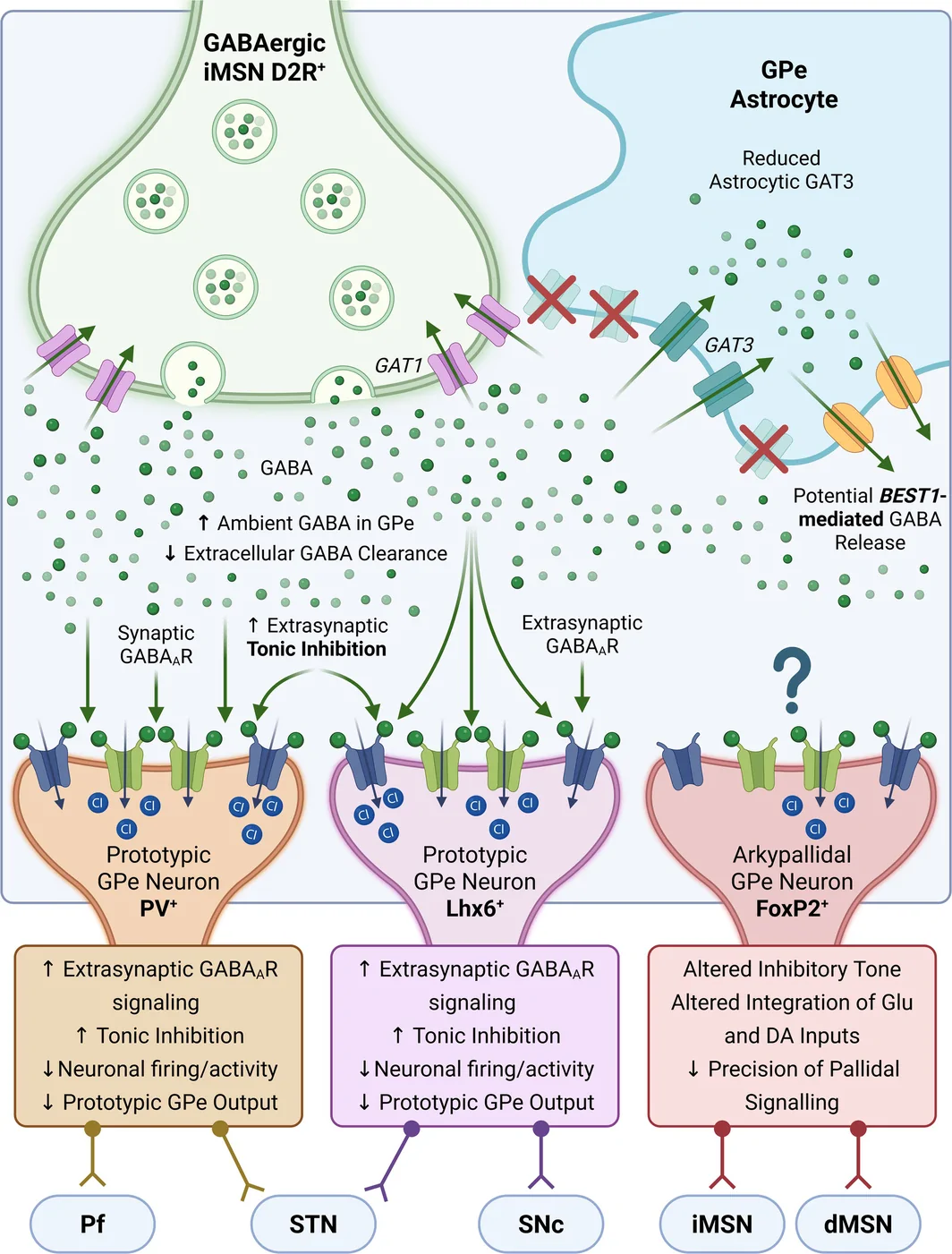
Reduced astrocytic GAT3 function may elevate ambient GABA and alter inhibitory signalling across GPe neuronal subtypes.

Taken together, these region‐specific effects suggest that alcohol‐induced changes in striatal inhibition, including shifts in ambient GABA tone, dopaminergic input and synaptic control of interneurons, may bias behavioural control. These alterations may modulate the dynamics of direct and indirect pathways across the DMS and DLS. Atypical tonic GABA signalling can disrupt the balance between goal‐directed and habitual actions by altering disinhibitory dynamics in striatal output pathways [6, 68]. As a result, both goal‐directed alcohol seeking and habitual, repetitive behaviours may be strengthened in parallel with prolonged alcohol exposure. The striatum translates these molecular‐level changes in inhibitory tone into behaviourally relevant patterns of action selection. Disruptions in this process link altered GABAergic signalling to excessive goal‐directed alcohol seeking and persistent use despite adverse consequences, as seen in AUD [11]. Thus, striatal GABAergic signalling forms a critical mechanistic bridge between alcohol‐induced inhibitory dysregulation and the progressive loss of behavioural flexibility that defines compulsive alcohol use.

## 3 Extracellular GABA Dysregulation and Receptor Dynamics During Acute and Chronic Alcohol Exposure

### 3.1 Stage‐Dependent Effects of Alcohol on GABAergic Signalling

Alcohol exposure produces stage‐dependent changes in GABAergic signalling as it progresses from controlled drinking to dependence and relapse. The central theme is that alcohol progressively dysregulates inhibitory control, shifting GABAergic signalling from acute enhancement to chronic adaptation. During acute exposure, alcohol enhances GABAergic transmission in several limbic regions, such as the amygdala and hippocampus, though these effects are not uniform across all areas. For example, in the central amygdala, alcohol at 11–66 mM enhances GABA_A_‐mediated IPSPs and IPSCs, with an EC50 of 20 mM. Decreased paired‐pulse facilitation and increased miniature IPSC frequency suggest both presynaptic and postsynaptic actions, which likely contribute to alcohol's acute anxiolytic and sedative effects and reinforce drinking behaviour [81].

In contrast, other regions display predominantly presynaptic effects: in cerebellar slices, 50 mM alcohol increases spontaneous IPSC frequency and tonic current, effects abolished by tetrodotoxin and associated with increased Golgi cell firing, indicating action potential–dependent GABA release rather than direct postsynaptic effects [82]. Alcohol‐enhanced GABA release occurs in many, but not all, brain regions and may depend on GPCR signalling, such as through CRF1 receptors in the central amygdala [83, 84]. Together, these findings show that increased inhibitory signalling reduces glutamatergic transmission and likely contributes to alcohol's anxiolytic and sedative effects, reinforcing acute drinking behaviour and helping sustain intake [16, 85, 86, 87]. As alcohol intake escalates, GABAergic enhancement continues alongside activation of mesolimbic reward pathways. This increases dopamine release, especially within the NAc, and reinforces voluntary alcohol use [76, 88]. With repeated binge or heavy drinking, compensatory neuroadaptations develop: GABA_A_ receptor subunits (e.g., α1 and α4) are downregulated and internalized, cortical GABA levels drop and hyperexcitability emerges [89]. These changes drive tolerance, requiring more alcohol to achieve the same level of inhibition, and help sustain heavier drinking and dependence by weakening inhibitory control [90]. Elevated extracellular GABA levels, due to impaired clearance and altered receptor signalling, are strongly implicated in the development and persistence of AUD [12, 86].

### 3.2 GABA_A_ Receptor Structure, Subunit Composition and Alcohol Sensitivity

This dysregulated GABAergic environment is especially important for GABA_A_ receptor signalling, which is consistently associated with alcohol‐seeking behaviours and genetic vulnerability to AUD [91]. GABA_A_ receptor structure helps explain why alcohol's effects vary across receptor subtypes and brain regions and why these differences matter for alcohol‐seeking behaviour. Many acute alcohol effects can be reproduced by positive allosteric modulators of GABA_A_ receptors, such as benzodiazepines, while chronic alcohol exposure leads to altered sensitivity to GABA_A_ receptor antagonists [92, 93]. Understanding alcohol‐induced changes in GABA_A_ receptor function, therefore, requires considering their structural and signalling properties. GABA_A_ receptors are pentameric chloride channels whose subunit composition determines their localization, kinetics and pharmacological sensitivity, shaping how alcohol affects their signalling and related behaviour [94, 95]. Typically, γ‐containing receptors mediate phasic inhibition, while δ‐containing receptors are responsible for tonic inhibition [87, 96]. Knockout studies of the predominant α1 and β2 subunits show that male mice lacking these subunits have reduced sensitivity to alcohol's sedative effects, as measured by loss of righting reflex, compared to wild‐type controls [97]. Several caveats are important when interpreting alcohol's actions at GABA_A_ receptors. First, most recombinant GABA_A_ receptor combinations only respond to alcohol at concentrations of 60 mM or higher [98]. Second, in native tissue, alcohol‐induced increases in inhibitory transmission are often presynaptic and depend on GPCR signalling [82, 83, 84]. Third, some postsynaptic effects may be indirect; for example, enhancing GABA‐stimulated tonic current requires PKCδ [99], and mice with anaesthetic‐insensitive β3(N265M) receptors show little change in acute alcohol sensitivity [100]. Alcohol thus enhances GABAergic signalling, but no single universal receptor‐level mechanism drives these effects, and the resulting behavioural outcomes likely reflect this layered regulation of inhibition.

Human genetic studies further implicate GABAA receptor subunit genes in AUD vulnerability, with GABRA2, the gene encoding the α2 subunit, showing the most consistent association. These genetic findings support a broad GABAergic contribution to alcohol vulnerability, even though individual variant effects are modest. Pedigree analyses from the Collaborative Study on the Genetics of Alcoholism (COGA) found modest associations between AUD‐related traits and several GABA_A_ receptor subunit genes, with parent‐of‐origin effects at the imprinted chromosome 15 cluster but not at chromosome five subunit genes [101]. Fine mapping of the chromosome 4 cluster in the same cohort revealed that several GABRA2 single‐nucleotide polymorphisms were linked to alcohol dependence and to β‐frequency EEG activity, an endophenotype reflecting cortical excitation‐inhibition balance [102]. A subsequent meta‐analysis of independent case–control and family‐based studies confirmed associations between GABRA2 and GABRG2 variants and alcohol dependence, with the strongest evidence for GABRA2. However, effect sizes for individual variants were modest [103]. Overall, these findings support a broad role for GABAergic signalling in alcohol vulnerability and suggest that genetic susceptibility and acute pharmacological sensitivity may be concentrated in different receptor subunits, potentially explaining differences in drinking‐related behaviour (see Table 3).

**TABLE 3:** Divergent genetic and functional contributions of GABA_A_ receptor subunits to AUD.

References	[104, 134]	[7, 102, 103, 135, 136, 137]	[105, 106, 117, 138]	[118, 119, 120, 121]	[122, 123]	[124]	[125]	[100]	[126, 127]	[128, 129]	[130]	[92, 99, 105, 107, 108]
**Association with AUD**	No consistent human genetic association.	Consistent genetic association with AUD across multiple studies.	No direct human AUD genetic association.	No direct AUD genetic association.	Weak association with alcohol sensitivity.	No direct human AUD genetic association.	No direct human AUD genetic association.	No direct human AUD genetic association.	Human population studies show GABRG1 haplotypes and SNPs were significantly. Associated with AUD.	Self‐Rating of the Effects of Alcohol Scale shows some correlations	Studies suggest may be involved in the risk for alcohol dependence and familial predisposition to developing AUD	No consistent human genetic association; strong behavioural relevance in animal models
**Alcohol‐mediated effects**	Chronic alcohol exposure reduces α1 expression and surface receptors in cortex/hippocampus.	Modulates ethanol sensitivity and reward‐related responses.	Upregulated following chronic alcohol exposure; contributes to increased extrasynaptic signalling.	Alcohol modulates α5‐containing receptors; implicated in cognitive effects of alcohol.	Inconsistent data regarding alcohol sensitivity. May mediate impaired motor coordination.	Limited data but gene may promote alcohol consumption through increased tonic inhibition.	Limited data but mRNA content is differentially regulated in alcohol‐dependant mouse models.	No major modulatory role relating to alcohol consumption. Limited data suggests role in mediating alcohol tolerance and withdrawal.	Genetic markers in 5′ *GABRG1* haplotype showed greater allelic, genotypic and haplotypic association with alcohol dependence.	Variation in gene and deletion show low alcohol sensitivity, high alcohol consumption and increased behavioural risk factors for addiction.	Limited studies on alcohol mediated effects.	Highly sensitive to low ethanol concentrations; mediates tonic inhibition; ethanol enhances tonic currents
**Gene**	*GABRA1*	*GABRA2*	*GABRA4*	*GABRA5*	*GABRA6*	*GABRB1*	*GABRB2*	*GABRB3*	*GABRG1*	*GABRG2*	*GABRG3*	*GABRD*
**GABA**_**A**_ **Subunit**	α1	α2	α4	α5	α6	β1	β2	β3	γ1	γ2	γ3	δ

### 3.3 δ‐Containing GABA_A_ Receptors and Alcohol‐Related Neuroadaptations

The δ subunit of GABA_A_ receptors has received particular attention because reports suggest that it is sensitive to low, behaviourally relevant alcohol concentrations. The central issue remains whether δ‐containing receptors are direct alcohol targets or are instead part of broader alcohol‐induced neuroadaptations. These receptors are localized extrasynaptically and frequently colocalize with α4 subunits in the striatum and thalamus [109, 110, 111, 112], making them plausible substrates for alcohol's effects on inhibitory tone and drinking behaviour. Reports that a low dose of alcohol (3 mM) enhances recombinant α4β3δ and α6β3δ receptors have not been consistently reproduced across laboratories and expression systems [113, 114, 115]. The concentration‐dependence of direct δ‐subunit modulation therefore remains unresolved, and alcohol‐induced increases in tonic current in these preparations may also reflect increased GABA release rather than direct receptor modulation [82].

In the cerebellum, mRNA expression of GABRD, the gene encoding the δ subunit, is significantly reduced in patients with AUD [116]. Consistent with altered δ‐subunit regulation, preclinical Drinking‐in‐the‐Dark (DID) with Intermittent Access (IA) models show that δ‐protein expression is highly plastic and dependent on the duration of alcohol exposure. During acute alcohol exposure, δ‐expressing receptors are upregulated, and selective loss of these δ‐GABA_A_Rs in PV interneurons increases binge drinking behaviour [107, 131]. In chronic alcohol exposure models, α1 receptors are significantly upregulated, while α4 receptors are downregulated in the cerebral cortex [132]. Together, these findings suggest that alcohol differentially modulates GABA_A_ receptor function depending on subunit composition, with distinct consequences for binge drinking and dependence‐related adaptation and behaviour.

Further supporting a key role for δ‐containing GABA_A_ receptors, δ‐subunit knockout models exhibit reduced alcohol consumption and attenuated withdrawal following chronic alcohol exposure, underscoring their potential as therapeutic targets [108]. Conditional deletion approaches further implicate δ‐GABA_A_Rs in alcohol‐related behaviours. In floxed GABRD mice, intra‐VTA AAV‐Cre‐GFP‐mediated knockdown of δ‐GABA_A_ receptors decreases binge‐like drinking in females [105]. Data also indicate that δ subunits colocalize to a lesser extent with α1 subunits, despite the typical associations of α1 subunits with extrasynaptic and δ subunits with synaptic domains, respectively. This less common α1/δ population may be functionally important: alcohol potentiates tonic currents mediated by these receptors at 20 and 30 mM, with the effect preserved in α4‐deficient mice and absent in δ‐deficient mice [106]. Whether this α1/δ population is functionally distinct from the α4/δ and α6/δ assemblies (for which low‐concentration alcohol sensitivity has not been consistently reproduced) remains an open question, but one that may warrant direct comparison in future work on drinking behaviour and withdrawal.

### 3.4 Baclofen and GABA_B_ Receptor Modulation of Alcohol Intake

AUD involves broad dysregulation across GABAergic systems, extending beyond GABA_A_ receptor subunit composition in binge‐like intake. Critically, dependence alters inhibitory control and GABA_B_ receptor modulation of dopaminergic neurons [133]. As alcohol use shifts from intermittent consumption to dependence, GABAergic function becomes increasingly disrupted, characterized by reduced inhibition and altered GABA_B_ receptor signalling. These adaptations may reinforce compulsive alcohol seeking through both positive and negative reinforcement mechanisms [104]. GABA_B_ receptors mediate inhibition by activating K+ channels, inhibiting voltage‐gated Ca2+ channels, and suppressing adenylyl cyclase activity [139]. Functional GABA_B_ receptors are heterodimers (GABA_B1_ and GABA_B2_) that reduce neuronal excitability and neurotransmitter release by these mechanisms [140, 141, 142, 143, 144]. Due to their downstream effects, GABA_B_ receptors are considered promising pharmacological targets for reducing alcohol‐related reinforcement. Baclofen, a GABA_B_ agonist, has been investigated as a potential therapy for reducing alcohol craving and intake [145, 146, 147]. Early studies demonstrated that 5 mg/kg baclofen eliminated alcohol‐stimulated locomotor activity in mice [148], implicating GABA_B_ activation in the attenuation of alcohol‐driven behaviours. Across two‐bottle‐choice paradigms, these findings show that baclofen most reliably reduces intake when repeated low‐to‐moderate dosing is used in animals with established high alcohol preference or restricted access. For example, R(+)‐baclofen (1.0–3.0 mg/kg, i.p.), administered as a single dose or daily for 4 days, reduced alcohol intake in selectively bred high‐alcohol‐drinking rats [149] and daily baclofen (3 mg/kg, i.p.) for 14 days reduced intake in alcohol‐preferring Long‐Evans rats without affecting total fluid intake [150].

Dose‐dependent reductions in alcohol intake have been observed in Sardinian alcohol‐preferring rats, with higher doses also increasing water intake, suggesting reduced behavioural selectivity at these levels [151]. In unselected animals, only higher baclofen doses (5.0 and 7.5 mg/kg) reduced alcohol consumption, while 2.5 mg/kg was ineffective [152]. Conversely, in mice, 2.5 mg/kg reduced intake in both control and high‐consuming groups [153], showing that baclofen's drinking‐related effects vary across phenotypes and doses while remaining centred on intake reduction.

Researchers observed a contrasting outcome under alternative conditions: when they administered a single high dose of baclofen (10 mg/kg, i.p.) to outbred Long‐Evans rats before every‐other‐day free‐choice sessions, the rats increased their absolute alcohol intake [154]. This finding links the administration schedule to the observed behavioural effect and demonstrates that identical pharmacological interventions can produce opposite outcomes depending on the dosing regimen. Dose also affects selectivity: in the Scheduled High Alcohol Consumption (SHAC) procedure, both 2.5 and 5.0 mg/kg baclofen decreased binge drinking, but the higher dose also reduced water intake and operant sucrose self‐administration, reflecting less specificity and broader effects on reward‐directed behaviours at higher doses [155]. Collectively, these studies indicate that baclofen's effect on drinking is modulated by phenotype, dose and administration schedule; repeated low‐to‐moderate dosing in animals with high preference or restricted access reduces intake, whereas high single doses may be less selective or even counterproductive.

Operant conditioning paradigms demonstrate that baclofen reduces alcohol‐directed responding across both fixed‐ and progressive‐ratio schedules, indicating decreased reinforcement and motivation [156, 157, 158, 159, 160]. These behavioural effects support the conclusion that GABA_B_ activation reduces alcohol reinforcement by lowering alcohol‐seeking behaviour, although questions remain regarding which animal populations are most sensitive to these effects.

Emerging evidence links GABA clearance mechanisms to compulsive drinking: rats with reduced GAT3 mRNA in the central, but not basolateral, amygdala persisted in drinking despite quinine adulteration and the availability of alternative reinforces. This finding connects diminished GABA clearance to resistance against aversive and competing behavioural challenges. Baclofen reduced intake in all rats but was particularly effective in those with low central amygdala GAT3, suggesting that GAT3 expression predicts treatment response under adulteration [145]. These results imply that diminished GAT3 may confer vulnerability to compulsive drinking while maintaining sensitivity to GABA_B_ modulation. However, the precise neural locus remains unclear, underscoring the need for further investigation into astrocytic GABA transport and ambient GABA signalling.

## 4 Astrocytic GAT3 and the Regulation of Ambient GABA Tone

### 4.1 GABA Transporters and Extracellular GABA Clearance

Beyond receptor dynamics, mechanisms regulating extracellular GABA critically shape the strength and duration of GABAergic signalling. While GABA_A_ and GABA_B_ receptors primarily mediate inhibitory transmission at synaptic and extrasynaptic sites, they are only part of a tightly regulated inhibitory system [161]. Because extracellular GABA is not enzymatically degraded, its clearance depends on GABA transporters [162]. Four main transporters in the solute carrier 6 (SLC6) family mediate this process: A1/GAT1, A13/GAT2, A11/GAT3 and A12/BGT1 [163, 164, 165]. This review centres on GAT3, whose astrocytic localization makes it a key regulator of ambient GABA tone and habit‐related behaviours in AUD. GAT1, the other major GABA transporter, primarily mediates rapid synaptic GABA clearance in neurons [41], whereas GAT3 is localized to distal astrocytic processes, where it regulates ambient extracellular GABA and tonic inhibition [41, 166]. Ultrastructural studies support this functional and anatomical distinction [167].

### 4.2 GAT3 Regulation of Extracellular GABA and Tonic Inhibition

This localization positions GAT3 to regulate extrasynaptic GABA levels and influence tonic inhibitory signalling, thereby shaping circuit output. Although GAT3's function is not fully characterized, accumulating evidence indicates that GAT3‐mediated GABA dynamics are critical for neuronal activity and behaviour [168, 169]. Blocking GAT3 reduces astrocytic GABA uptake and increases extracellular GABA, which may trigger a compensatory increase of transporter expression or activity [164, 170]. The impact of GAT3 depends strongly on network activity: In vivo studies show that GAT1 primarily regulates extracellular GABA under baseline conditions, but astrocytic GAT3 becomes increasingly important during high neuronal activity, limiting GABA spillover, tonic inhibition and circuit output [38]. Thus, alcohol‐induced changes in GAT3 expression and function can disrupt GABA homeostasis and alter behaviour in AUD‐relevant circuits.

### 4.3 GAT3 Dysfunction and Alcohol‐Related Behavioural Inflexibility

To clarify how transporter‐specific effects relate to AUD vulnerability and progression, both genetic and functional evidence must be considered. Table 4 summarizes findings for the four SLC6 family transporters: genetic support is strongest for GAT1, while GAT2 and BGT1 show limited central expression. GAT3 lacks conclusive GWAS or QTL association with AUD but demonstrates the strongest functional and translational evidence among the four. The absence of AUD‐specific association does not mean SLC6A11 variation is silent, as GAT3 polymorphisms are linked to febrile seizures and temporal lobe epilepsy [174]. This dissociation underlines the focus on GAT3 as the transporter most relevant to the review's central argument. Alcohol‐induced neural toxicity may impair GAT3 function, weakening behavioural flexibility and reducing disengagement from habitual alcohol seeking (Figure 1) [175]. Behavioural studies support this link: disrupted inhibitory regulation impairs flexibility in alcohol choice, as shown in rat and human tissue in a single integrated study [173]. In an exclusive choice paradigm, some rats preferred alcohol over a high‐value natural reward and displayed addiction‐like traits, including persistent seeking despite negative consequences. This behavioural profile was associated with selective downregulation of GAT3 in the amygdala. Moreover, experimental knockdown of GAT3 shifted reward preference towards alcohol, providing a direct link between impaired GAT3‐mediated GABA clearance and alcohol‐biased decision‐making. Consistent with these preclinical findings, the same study found reduced GAT3 expression in post‐mortem central amygdala tissue from individuals with alcohol dependence compared with controls [173]. Together, these findings implicate impaired astrocytic GABA transport in maladaptive alcohol seeking and identify the amygdala as a key site of this effect. More broadly, they suggest that reduced GABA clearance and the resulting increase in tonic inhibition can alter the function of neural circuits that regulate behavioural choice, thereby favouring maladaptive behavioural patterns.

**TABLE 4:** Divergent genetic and functional contributions of GABA transporters to AUD.

GABA transporter	Gene	Association with AUD	References
GAT1	*SLC6A1*	Genetic variation in *SLC6A1* has been associated with risk for alcoholism across multiple populations, suggesting a role in modulating synaptic GABA and vulnerability to AUD.	[171]
GAT2	*SLC6A13*	No direct evidence linking *SLC6A13* variants to AUD; primarily expressed in peripheral tissues and liver, with limited central role in addiction‐related circuitry.	[172]
GAT3	*SLC6A11*	No conclusive GWAS/QTL association with AUD; however, strong functional and translational evidence shows reduced GAT3 expression in the amygdala of alcohol‐preferring rodents and individuals with alcohol dependence, implicating impaired GABA clearance in compulsive alcohol seeking.	[173]
BGT1	*SLC6A12*	No established association with AUD; limited CNS expression and minimal evidence linking this transporter to addiction‐related behaviours.	[172]

Chronic alcohol does not shift astrocytic GABA transport in a single direction. During withdrawal from chronic intermittent alcohol exposure, the central amygdala exhibits increased IPSC frequency and the emergence of a tonic GABA current, with a GAT3‐sensitive component unmasked only after alcohol exposure. Astrocytes respond by selectively upregulating GAT3, increasing astrocyte‐synapse proximity and maintaining increased apposition of GAT3‐containing processes to synapses beyond acute withdrawal in both sexes [176]. This upregulation likely represents a homeostatic response to sustained GABAergic load, not a contradiction of the reductions described above, which reflect baseline transport capacity in vulnerable animals.

Notably, neither viral nor pharmacological manipulation of GAT3 in the central amygdala alters dependence‐escalated, aversion‐resistant or postdependent alcohol drinking [176]. These findings suggest that the behavioural effects of GAT3 depend on the specific outcome examined: GAT3 levels influence the choice between alcohol and a competing reward but appear to have little effect on alcohol consumption once dependence is established (Figure 1).

### 4.4 Astrocytic Calcium Signalling, Plasticity and Perseverative Behaviour

Pharmacological studies using the GAT3‐selective inhibitor SNAP‐5114 show that GAT3 regulates extracellular GABA and tonic inhibition. Altered GAT3 function can directly affect activity across dorsomedial and dorsolateral striatal circuits, shifting the balance between goal‐directed and habitual control [42, 173]. In striatal circuits, changes in MSN excitability and output influence whether behaviour remains flexible and outcome‐sensitive or becomes rigid and habitual [63]. Because GAT3 is primarily localized to astrocytes, it may serve as a key modulator of tonic inhibition and, in turn, shape circuit excitability and disinhibitory dynamics. Through these mechanisms, GAT3 influences the expression of flexible versus habitual behaviours.

Beyond tonic inhibition, GAT3 may provide a mechanistic link between astrocytic control of extracellular GABA and broader circuit plasticity. Astrocytic dysfunction may therefore disrupt synaptic plasticity, a process frequently altered in AUD [177, 178]. Specific astrocytic populations can regulate perseverative behaviour through modulating intercellular calcium signalling, and disruptions in this signalling may contribute to compulsive actions relevant to AUD [179, 180, 181, 182]. Perseverative behaviours are inflexible or inappropriate action patterns persistently repeated despite changing demands or outcomes [183, 184]. Human genetic evidence also implicates GABA transporter loss in this behavioural domain: A 3p25.3 microdeletion spanning SLC6A1 and SLC6A11 produces intellectual disability, epilepsy and stereotypic behaviour, although deletion of both genes precludes attributing the phenotype to GAT3 alone [185]. Post‐mortem brain analysis in OCD, a disorder associated with perseverative behaviours [186, 187], revealed downregulation of CRYM, encoding the astrocyte‐associated protein μ‐crystallin. Consistent with this, *crym* knockout mice exhibit increased perseverative behaviours. In the striatum, crym+ astrocytes account for about 49% of all astrocytes, with a regionally biased distribution. They are minimal in the dorsolateral region and enriched in ventral and central regions, suggesting a contribution to circuits involved in behavioural flexibility. Interestingly, astrocyte‐specific reduction of calcium signalling enhances both compulsive and perseverative behaviours [181] and may be accompanied by compensatory increases in GAT3 expression. In this state, astrocytes may become less able to dynamically regulate the synaptic environment and instead shift towards increased basal GABA clearance. Rather than supporting temporally precise, activity‐dependent modulation, this compensatory state may favour more sustained regulation of extracellular GABA, shifting the system towards tonic modulation and promoting inflexible, persistent action patterns.

### 4.5 From Habit Formation to Failed Adaptive Disengagement

Importantly, although GAT3 is often discussed as a modulator of the balance between goal‐directed and habitual control, emerging evidence suggests a complementary role in regulating the persistence of behavioural strategies when contingencies change. In this framework, GAT3‐mediated regulation of extracellular GABA and tonic inhibition may influence not only whether behaviour is goal‐directed or habitual, but also whether maladaptive actions are abandoned or persist despite adverse outcomes. Elevated tonic inhibition within corticostriatal circuits could reduce sensitivity to outcome devaluation and aversive feedback, maintaining action policies even when the expected outcome is no longer beneficial. Thus, GAT3 may help determine whether behavioural strategies are flexibly updated or persist, reinforcing the review's central focus on adaptive disengagement.

This distinction is important because maladaptive persistence does not necessarily reflect habit strength alone. Perseverative alcohol seeking may be better understood not simply as overreliance on habit systems, but as a failure of adaptive disengagement. Rather than only reflecting a transition from goal‐directed to habitual control, AUD may involve goal‐directed representations that fail to update, leading to behaviour driven by outdated value assessments despite negative consequences. In this context, GAT3 dysfunction may link impaired astrocytic GABA clearance to maladaptive tonic inhibitory tone and persistent alcohol seeking, highlighting astrocyte‐mediated GABA regulation as a key mechanism underlying behavioural inflexibility in AUD.

## 5 Discussion

In this review, we argue that alcohol‐induced alterations in GABAergic signalling within striatopallidal circuits are central to AUD because they shape behavioural flexibility and reinforcement processes underlying compulsive drinking [1, 5, 6]. By synthesizing findings across molecular, cellular and circuit levels, we identify GABAergic adaptations as a key mechanism driving maladaptive alcohol‐related behaviours [3, 23, 38]. We emphasize the need to examine these adaptations in a circuit‐, cell‐type‐ and context‐specific manner to clarify how alcohol reshapes behaviour [6, 7, 9, 50]. Translating these mechanistic insights into effective clinical interventions remains a significant challenge [14, 145]. Our discussion highlights the value of integrating preclinical models with innovative clinical approaches, including identifying reliable biomarkers and developing targeted therapies, to advance more precise strategies for treating AUD [86, 87].

Building on this foundation, we propose a unifying model in which astrocytic GAT3 dysfunction shapes inhibitory tone and behavioural flexibility in AUD [38, 39, 42]. We synthesize evidence across molecular, synaptic and circuit levels to support this model [23, 24, 38, 42]. While preclinical evidence strongly implicates GAT3 and astrocyte‐mediated GABA clearance in maladaptive alcohol seeking and habit persistence [145], direct demonstration in human tissue and the development of specific therapeutic interventions remain key priorities. Future research should therefore clarify these causal relationships in human‐relevant models and develop compounds that selectively modulate astrocytic GABA transport [39, 42].

Translating these mechanistic insights to clinical application presents unique opportunities and challenges [14, 145]. Although no clinical trials have yet targeted astrocytic GABA transporters such as GAT3 in AUD, astrocyte‐directed therapies are gaining momentum across central nervous system disorders [23, 42]. In the context of GABAergic modulation, some studies suggest that manipulating astrocytic GAT3 can reduce compulsive behaviours, while others report minimal impact or compensatory changes in other neurotransmitter systems [38, 145]. These conflicting findings underscore the complexity of targeting astrocyte‐mediated neurotransmitter clearance and its effects on compulsive behaviour.

Looking ahead, gene therapy and small‐molecule approaches to modulate astrocyte function may become feasible strategies for treating behavioural inflexibility in AUD and related conditions, including by targeting astrocytic GABA clearance [145, 161]. However, the development of astrocyte‐targeted therapies for AUD presents several challenges [23, 38]. These include potential off‐target effects on other cell types or neurotransmitter systems, difficulty achieving selective drug delivery across the blood–brain barrier and regulatory hurdles that often accompany novel gene‐ or cell‐based interventions [42]. Addressing these challenges will require advances in drug design, delivery technologies and rigorous safety evaluation [86, 87].

These advances hold promise not only for AUD, but for a broader class of disorders characterized by compulsive, inflexible behaviour [15, 50]. Nevertheless, further research directly comparing these approaches, as well as integrating alternative theories regarding the interplay between astrocytic and neuronal signalling, will be critical for optimizing therapeutic strategies that improve behavioural flexibility [13, 87].

Broadening the discussion beyond AUD, the mechanistic insights presented here may also enhance our understanding of other compulsive disorders and the fundamental principles underlying habit formation and behavioural flexibility [9, 15, 50]. We must acknowledge several limitations, as they influence both how we interpret these findings and their translational potential. Most evidence summarized in this review is derived from preclinical animal models, which may not fully capture the complexity of human AUD [1, 38]. Differences in neuroanatomy, compensatory mechanisms and behavioural repertoires between species can limit the translational value of animal findings [13, 23]. Additionally, current technical limitations in assessing GABAergic function in vivo, along with the scarcity of human data on astrocytic GAT3, present significant obstacles to directly validating findings from preclinical models [39, 42]. Moreover, while this review focuses on GABAergic mechanisms, AUD is a multifactorial disorder involving multiple neurotransmitter systems, genetic backgrounds and environmental factors, which may also influence treatment outcomes [103, 175]. Comorbidity with other psychiatric and neurological disorders, such as anxiety, depression and neurodegenerative diseases, adds further complexity [175]. GABAergic and astrocytic mechanisms implicated in AUD may also contribute to, or be influenced by, these comorbid conditions, potentially affecting both disease progression and treatment efficacy [14, 175]. In addition, sex differences and individual variability, including genetic, epigenetic and environmental influences, may significantly affect GABAergic signalling and the efficacy of targeted interventions. Few studies have systematically addressed these factors, but emerging evidence suggests that biological sex and individual differences can shape both vulnerability to AUD and responses to pharmacological treatment [102, 103].

As research continues to uncover the complex astrocyte‐neuron interactions underlying maladaptive behaviour, it will be important to integrate findings from diverse methodologies and models and to validate them in clinical settings [23, 38]. Another important consideration is how the developmental stage and aging influence GABAergic mechanisms in AUD [90, 175]. Evidence suggests that the impact of alcohol on GABAergic signalling, as well as the efficacy of potential interventions, may vary across the lifespan, with adolescence and older age potentially representing periods of heightened vulnerability or altered response [90]. Future studies should therefore examine how age and developmental trajectories modulate GABAergic adaptations and treatment outcomes [175]. Novel circuit‐based and astrocyte‐targeted therapies could reshape the treatment landscape for AUD and related conditions. Still, careful consideration of these limitations will be essential for successful translation and improved behavioural outcomes [13, 87].

To further illustrate the circuit‐level implications of astrocytic dysfunction, it is helpful to focus on the external segment of the globus pallidus (GPe) (Figure 2). Among multiple brain regions, the GPe integrates input from both striatal regions responsible for goal‐directed and habitual control and exhibits among the highest GAT3 expression in the brain [188, 189]. This unique position makes the GPe especially vulnerable to astrocytic dysfunction and impaired GABA clearance [38, 39, 42]. Within the GPe, reduced GAT3‐mediated uptake may elevate extracellular GABA by limiting astrocytic clearance of inhibitory inputs from iMSNs and local pallidal terminals [38, 42]. This increase in ambient GABA enhances tonic inhibition via extrasynaptic GABA_A_ receptors and suppresses prototypic GPe neurons, especially PV+ and Lhx6+ subpopulations [42]. The resulting reduction in GPe output to the subthalamic nucleus (STN) increases STN activity, which may disrupt stop‐related signalling in cortico‐basal ganglia circuits and promote inflexible action selection and habitual responding [6, 7, 50]. Taken together, as summarized in Figure 2, this model suggests that astrocytic GAT3 dysfunction in the GPe could lead to excessive ambient GABA, reduced prototypic GPe output and secondary STN disinhibition, creating a circuit state that favours maladaptive action selection, habitual alcohol seeking and behavioural inflexibility [42, 145]. Thus, astrocytic GAT3 serves as a circuit‐level regulator of behavioural flexibility in AUD by linking impaired GABA clearance to habitual alcohol seeking [38, 42, 145].

In summary, although preclinical studies provide a strong mechanistic foundation for understanding GABAergic dysregulation in AUD [13, 38, 42, 86], further work is warranted to establish the translational relevance of these findings in humans [14, 145]. Among the most urgent next steps, future research should directly validate the role of astrocytic GAT3 and GABA clearance mechanisms in human tissue and in vivo models using advanced imaging, single‐cell transcriptomics and functional assays [39, 42]. Emerging technologies, such as optogenetics, spatial transcriptomics and single‐cell multiomics, offer unprecedented opportunities to unravel circuit‐ and cell‐specific mechanisms underlying GABAergic signalling and astrocyte function in AUD [42, 87]. The development of reliable biomarkers, such as PET imaging ligands or biochemical assays for astrocytic GABA transporter activity, remains a top priority for monitoring target engagement in both animal models and clinical cohorts [39, 42]. Experimental approaches such as CRISPR‐based gene editing, chemogenetic modulation and in vivo two‐photon imaging could yield deeper insights into cell‐type‐ and circuit‐specific mechanisms [42, 87].

On the clinical side, innovative trial designs that incorporate patient stratification based on neurobiological markers, genetics or behavioural phenotypes will be crucial for identifying subpopulations most likely to benefit from GABAergic or astrocyte‐targeted interventions [102, 103, 175]. Interdisciplinary collaborations among neuroscientists, clinicians, bioengineers and computational biologists will accelerate the translation of mechanistic discoveries into practical therapies [87]. As the field advances towards clinical translation, it will also be important to consider ethical and societal implications, such as equitable access to advanced therapies and the responsible use of technologies like gene editing [87]. Addressing these issues proactively will help ensure that innovations in GABAergic and astrocyte‐targeted interventions benefit diverse populations and adhere to ethical standards. Bridging these mechanistic and translational gaps will be critical to determining whether modulating GABAergic signalling can restore adaptive behavioural control and ultimately provide more precise and effective therapeutic strategies for individuals with AUD [86, 87].

This schematic illustrates the central question of whether reduced astrocytic GABA transporter 3 (GAT3) function impairs GABA clearance in the globus pallidus externus (GPe), thereby disrupting GPe–subthalamic nucleus (STN) regulation and altering basal ganglia output. Under normal conditions, astrocytic GAT3 facilitates GABA clearance and supports glutamine cycling, thereby maintaining low ambient GABA levels, physiological tonic inhibition and GPe neuronal firing. The figure proposes that reduced GAT3 function may impair astrocytic GABA clearance and glutamine cycling, increasing ambient GABA and enhancing extrasynaptic GABA_A_ and GABA_B_ receptor signalling. This would elevate tonic inhibition of GPe neurons, potentially reduce GPe firing and disinhibit STN activity, thus disrupting GPe–STN control of cortical and striatal information flow. Impaired basal ganglia output regulation resulting from reduced astrocytic GAT3 may contribute to maladaptive behavioural control in addiction. In the schematic, green spheres represent GABA (different sizes are used for illustrative and artistic purposes to convey depth), green arrows indicate the flow of GABA, light blue spheres represent glutamate, light blue lines indicate the flow of glutamate, dark blue lines depict the flow of chloride ions into GABA channels, and pink spheres represent potassium. Created with BioRender.com.

This schematic illustrates how impaired astrocytic GABA transporter 3 (GAT3) function may increase extracellular GABA tone within the globus pallidus externus (GPe). GABA released from indirect‐pathway medium spiny neuron (iMSN; D2R+) terminals is normally cleared by transporters, including astrocytic GAT3. When the GAT3 function is reduced, extracellular GABA clearance decreases, ambient GABA increases and spillover beyond the synaptic cleft may occur. Elevated extracellular GABA may then enhance signalling through extrasynaptic GABA_A_ receptors, increase tonic inhibition and reduce neuronal firing/activity in prototypic PV+ and Lhx6+ GPe neurons. This shift may weaken prototypic GPe output to downstream basal ganglia targets. In parallel, altered inhibitory tone may disrupt the integration of glutamatergic and dopaminergic inputs across GPe neuronal subtypes, including FoxP2+, PV+ and Lhx6+ neurons, thereby reducing the precision of pallidal signalling. BEST1‐mediated astrocytic GABA release may also contribute to ambient extracellular GABA and sustain extrasynaptic receptor activation. Green spheres represent GABA, and arrows indicate the direction of GABA movement or signalling. Arrows within GABA_A_ receptors represent receptor activation. Greater receptor activation is depicted for PV+ and Lhx6+ prototypic neurons. In contrast, reduced activation is observed in FoxP2+ neurons to indicating a less direct or less strongly supported predicted effect of elevated ambient GABA. These differences are conceptual and are not intended to represent quantitative receptor number, receptor occupancy or measured effect size. GAT1 and GAT3 are shown as transporter pathways, and BEST1 is depicted as a potential astrocytic GABA‐release pathway. Together, the model proposes that reduced astrocytic GAT3 function increases ambient GABA, drives tonic inhibition and alters pallidal signalling, thereby contributing to disrupted regulation of basal ganglia circuits and maladaptive behavioural control in addiction. Created with BioRender.com.

## Author Contributions

Nathan Jones and Hannah Aranda contributed equally to this work and are co‐first authors. Both contributed to the conceptualisation, literature review, drafting, and revision of the manuscript. Nathan Jones created all original figures using BioRender. Humza Haroon contributed to the literature review and critical revision of the manuscript. Doo‐Sup Choi supervised the work, contributed to the conceptualisation and critical revision of the manuscript, and served as the corresponding author. All authors reviewed and approved the final manuscript.

## Funding

This research was supported by the Samuel C. Johnson for Genomics of Addiction Program at the Mayo Clinic, the Ulm Foundation and the National Institute of Health (AA029258 and AA031167).

## Disclosure

Figures adapted from the Allen Mouse Brain Atlas and other published sources are reproduced or adapted in accordance with the applicable permissions, licences and citation requirements. Any required permissions have been obtained by the authors.

## Ethics Statement

The authors confirm that this manuscript is an original work, has not been published previously and is not under consideration for publication elsewhere. All authors have approved the submitted version of the manuscript and agree with its submission to *Addiction Biology*. The manuscript complies with the journal's ethics and integrity policies.

## Conflicts of Interest

The authors declare no conflicts of interest.

## Data Availability

This article is a review of previously published studies. No new datasets were generated or analysed during the preparation of this manuscript. All information discussed is available through the cited literature.
